# Spinal cord NLRP1 inflammasome contributes to dry skin induced chronic itch in mice

**DOI:** 10.1186/s12974-020-01807-3

**Published:** 2020-04-20

**Authors:** Jun-Juan Fan, Bo Gao, Ao-Qi Song, Ya-Jing Zhu, Jun Zhou, Wei-Zu Li, Yan-Yan Yin, Wen-Ning Wu

**Affiliations:** 1 Department of Pharmacology, School of Basic Medical Sciences, Anhui Medical University, Hefei, 230032 People’s Republic of China; 2 Key Laboratory of Anti-inflammatory and Immunopharmacology, Anhui Medical University, Hefei, 230032 People’s Republic of China; 3 Department of Pharmacy, Xi’an Chest Hospital, Shaanxi University of Chinese Medicine, Xi’an, 710100 People’s Republic of China

**Keywords:** NLRP1 inflammasome, TRPV1, Chronic itch, Dry skin, Spinal cord

## Abstract

**Background:**

Dry skin itch is one of the most common skin diseases and elderly people are believed to be particularly prone to it. The inflammasome has been suggested to play an important role in chronic inflammatory disorders including inflammatory skin diseases such as psoriasis. However, little is known about the role of NLRP1 inflammasome in dry skin-induced chronic itch.

**Methods:**

Dry skin-induced chronic itch model was established by acetone-ether-water (AEW) treatment. Spontaneous scratching behavior was recorded by video monitoring. The expression of nucleotide oligomerization domain (NOD)-like receptor protein 1 (NLRP1) inflammasome complexes, transient receptor potential vanilloid type 1 (TRPV1), and the level of inflammatory cytokines were determined by western blot, quantitative real-time PCR, and enzyme-linked immunosorbent assay (ELISA) kits. Nlrp1a knockdown was performed by an adeno-associated virus (AAV) vector containing Nlrp1a-shRNA-eGFP infusion. H.E. staining was used to evaluate skin lesion.

**Results:**

AEW treatment triggers spontaneous scratching and significantly increases the expression of NLRP1, ASC, and caspase-1 and the levels of IL-1β, IL-18, IL-6, and TNF-α in the spinal cord and the skin of mice. Spinal cord Nlrp1a knockdown prevents AEW-induced NLRP1 inflammasome assembly, TRPV1 channel activation, and spontaneous scratching behavior. Capsazepine, a specific antagonist of TRPV1, can also inhibit AEW-induced inflammatory response and scratching behavior. Furthermore, elderly mice and female mice exhibited more significant AEW-induced scratching behavior than young mice and male mice, respectively. Interestingly, AEW-induced increases in the expression of NLRP1 inflammasome complex and the levels of inflammatory cytokines were more remarkable in elderly mice and female mice than in young mice and male mice, respectively.

**Conclusions:**

Spinal cord NLRP1 inflammasome-mediated inflammatory response contributes to dry skin-induced chronic itch by TRPV1 channel, and it is also involved in age and sex differences of chronic itch. Inhibition of NLRP1 inflammasome may offer a new therapy for dry skin itch.

## Background

Itch, also known as pruritus, is a kind of disorder gives rise to unpleasant sensation that leads to scratching behavior [1–3]. Itch lasting more than 6 weeks is defined as chronic itch [4]. Many dermatologic and systemic diseases such as allergic contact dermatitis (ACD), atopic dermatitis (AD), psoriasis, post-herpetic itch, and chronic kidney disease are accompanied with chronic itch [5], which can be debilitating and significantly reduce the quality of life. Though the sensitization of itch signaling pathways is considered as a potential mechanism for chronic itch [6–8], little is known about the pathophysiological process of this disorder.

Inflammasome is a multi-protein complex that promotes the maturation of pro-inflammatory caspases, particularly caspase-1 [9, 10]. Active caspase-1 is a key enzyme conducting the cleavage of pro-IL-1β and pro-IL-18 into biologically active forms of IL-1β and IL-18 [11–13]. Inflammasomes have been suggested to play an important role in chronic inflammatory conditions including inflammatory skin diseases such as psoriasis [14]. The nucleotide-binding oligomerization domain-like receptor pyrin domain-containing 1 (NLRP1) inflammasome is the first characterized inflammasome and composed of NLRP1, an adaptor known as apoptosis-associated speck-like protein containing a caspase-activating recruitment domain (ASC), and caspase-1 [10, 15, 16]. Previous studies showed that NLRP1 inflammasome is related to many nervous system diseases such as traumatic brain injury (TBI), spinal cord injury (SCI), and epilepsy [17–20]. Also, NLRP1 inflammasome is the predominant inflammasome sensor in human skin [21] and has been reported to be involved in the pathological processes of nociception [22]. Furthermore, genome-wide association studies suggest that the NLRP1 haplotype appears in autoimmune diseases associated with psoriasis and vitiligo, and has an effect on skin-specific immune responses [14, 23, 24]. However, the role of NLRP1 inflammasome in chronic itch remains unclear.

Age and gender are considered as two principal risk factors in many diseases such as cardiovascular disease, metabolic diseases, neurodegenerative disorders and ischemic stroke [25–29]. Age and gender also play important roles in the development of itch. The elderly are particularly prone to dry skin itch especially in the case of irritant contact dermatitis [30, 31]. About 7.3–37.5% patients with chronic itch are over 60 years old [32]. Meanwhile, recent research demonstrated that women usually show higher itching intensity than men, and women also have more psychological triggers for itch than men [33, 34]. However, little is known about the mechanism underlying the age and gender differences of itch especially chronic itch. Therefore, in this study, we mainly investigated the involvement of NLRP1 inflammasome in dry skin itch and explored whether NLRP1 inflammasome is related to age and gender differences in the chronic itch model.

## Methods

### Animals

Male C57BL/6 mice (aged 6–10 weeks and 20 months) and young adult mice (6–10 weeks) of both sexes were obtained from the Experimental Animal Center of Anhui Medical University. They were kept in a controlled environment with a temperature of 22 ± 2 °C and humidity of 60% under a 12 h light/dark cycle. Food and water were available ad libitum. All animal procedures were approved by the Committee for Experimental Animal Use and Care of Anhui Medical University.

### Chemicals

Primary antibodies of NLRP1, TRPV1, caspase-1, IL-6, and TNF-α were purchased from Abcam (San Francisco, CA, USA). Primary antibodies of ASC, IL-1β, and IL-18 were purchased from Santa Cruz Biotechnology (Santa Cruz, CA, USA). Horseradish peroxidase-conjugated secondary antibodies were obtained from Santa Cruz Biotechnology (Santa Cruz, CA, USA). Capsazepine was obtained from Sigma-Aldrich (St. Louis, MO). Other general agents were commercially available.

### Dry skin itch model

To experimentally induce dry skin, we treated the nape of mice with acetone-ether-water (AEW) as previously reported [35]. The hair of the nape was shaved at least 3 days before the start of AEW treatment. Then, we treated the back skin by cotton (2 × 2 cm^2^) soaked with a 1:1 mixture of acetone and diethyl ether (AE) which was laid upon the shaved area for 20 s. After AE treatment, cotton soaked with distilled water was laid upon the same area for 30 s immediately. The treatment was performed twice daily (9:00 and 17:00) for 5 days. Cotton soaked with distilled water was laid on the shaved area for 50 s in the control group.

### Behavioral analysis

Mice were put individually into plastic chambers (14 × 18 × 12 cm) on an elevated metal mesh floor and allowed 30 min for habituation. Then, the spontaneous scratching was video recorded with experimenters kept out of the room for 1 h in the morning on day 6 and the total number of scratches was counted blindly. A scratch was counted when a mouse lifted the hind paw to scratch the shaved region and returned the paw to the floor or mouth for licking [36].

### Western blotting

Dissected spinal cord tissues were homogenized in lysis buffer containing 50 mM Tris-base (pH 7.4), 100 mM NaCl, 1% NP-40, 10 mM EDTA, 20 mM NaF, 1 mM PMSF, and protease inhibitors. After being lysed for 30 min on ice, samples were centrifuged at 12,000×*g* at 4 °C for 15 min. Supernatant was separated, and protein concentration was determined using the BCA protein assay kit (Pierce Biotechnology, Inc, Rockford, IL, USA). Protein samples (30 μg) were separated by 10–12% SDS-polyacrylamide gels and then transferred onto a PVDF membranes (Millipore). After blocking with 5% nonfat milk in Tris-buffered saline containing 0.1% Tween-20 (TBST) for 1 h at room temperature and rinsing, membranes were incubated with different primary antibodies (anti-NLRP1, anti-caspase-1, anti-IL-1β, anti-IL-6, and anti-TNF-α, 1:800 dilution; anti-ASC and anti-IL-18, 1:200 dilution) overnight at 4 °C. After washing, and followed by incubation with horseradish peroxidase-conjugated secondary antibodies (1:10 000 dilution) in TBST with 1% nonfat milk for 1 h at room temperature, the membranes were reacted with enhanced chemiluminescence reagents (Amersham Pharmacia Biotech, Inc., Piscataway, NJ, USA) for 5 min and were visualized using chemiluminescence detection system (Bioshine, Shanghai, China).

### Quantitative real-time PCR analysis

Total RNA was extracted from the spinal cord using TRIzol reagent (Invitrogen, USA) following the manufacturer’s instructions. cDNA synthesis was performed using a PrimeScriptfist Strand cDNA Synthesis Kit (Takara Biotechnology). PCR amplification of cDNA was performed by standard methods. The following specific primers were used: NLRP1 (forward: 5-TGGCACATCCTAGGGAAATC-3, reverse: 5-TCCTCACGTGACAGCAGAAC-3); ASC (forward: 5-GTCACAGAAGTGGAC GGAGTG-3, reverse: 5-CTCATCTTGTCTTGGCTGGTG-3); Caspase-1 (forward: 5-CGTGGAGAGAAACAAGGAGTG-3, reverse: 5-AATGAAAAGTGAGCCCCT GAC-3); β-actin (forward: 5-ACAACCTTCTTGCAGCTCCTC-3, reverse: 5-CTGA CCCATACCCACCATCAC-3). The fluorescent signals were collected during extension stage, and Ct values of the sample were calculated and relative transcript levels were analyzed by 2^−ΔΔCt^ method.

### Enzyme-linked immunosorbent assay (ELISA)

The protein samples were extracted and protein concentration was determined as described above. The levels of maturated IL-1β, maturated IL-18, IL-6, and TNF-α in the spinal cord were measured by commercial ELISA kits (R&D Systems, Minneapolis, MN, USA) according to the manufacturer’s protocol.

### Virus injection

To silence spinal cord Nlrp1, adeno-associated virus (AAV) vectors containing Nlrp1a-shRNA (can clear whole Nlrp1) or control-shRNA (Hanbio, Shanghai, China) was employed. In brief, Nlrp1a-shRNA or control-shRNA was cloned into pHBAAV-U6-MCS-CMV-eGFP (AAV2/9, 1.0 × 10^12^ TU/ml) and confirmed by sequencing. The recombinant plasmids were treated using a triple-transfection, helper-free method, and purified. The sequences for scrambled control-shRNA and Nlrp1a-shRNA were 5′- TTCTCCGAACGTGTCACGTAA-3′ and 5′-CAGCTAGAGAGGAACTTGAAGCT AA-3′, respectively. For the virus infusion, 2 μl control-shRNA or Nlrp1a-shRNA were intrathecally (i.t.) injected into the L5–L6 intervertebral space of mice (6 weeks old) at a rate of 0.2 μl/min using a 5 μl-Hamilton syringe connected to a 30-gauge needle. The flick of the tail was considered as an indicator of a successful i.t. administration. After injection, mice recovered for 28 days, to ensure viral expression and recombination. Fluorescence microscopy and western blotting could observe the effects of transfection in vivo. The AEW protocol was implemented 4 weeks after the i.t. injection. And the spontaneous scratching of the mice in each group was video recorded on day 6 after AEW treatment.

### Histological assay

After the behavioral observation, skin specimens were dissected immediately after animals were killed and fixed in 4% paraformaldehyde. Paraffinized skins were cut into 5 μm sections using microtome and were stained with hematoxylin and eosin (H.E.). The morphology of the epidermal layers was examined by light microscope (Olympus IX71, Tokyo, Japan). The thickness of nucleated epidermal layers was measured using ImageJ software from × 20 bright-field images taken at two or three randomly selected fields per section.

### Statistical analysis

All data were analyzed with the statistical program SPSS 17.0 (Chicago, IL, USA). Data are expressed as means ± SEM. Unpaired two-tailed Student’s *t* test or one-way analysis of variance (ANOVA) was used to evaluate differences. *P* < 0.05 was considered statistically significant.

## Results

### NLRP1 inflammasome is activated in AEW-induced pruritic mice

**Fig. 1:**
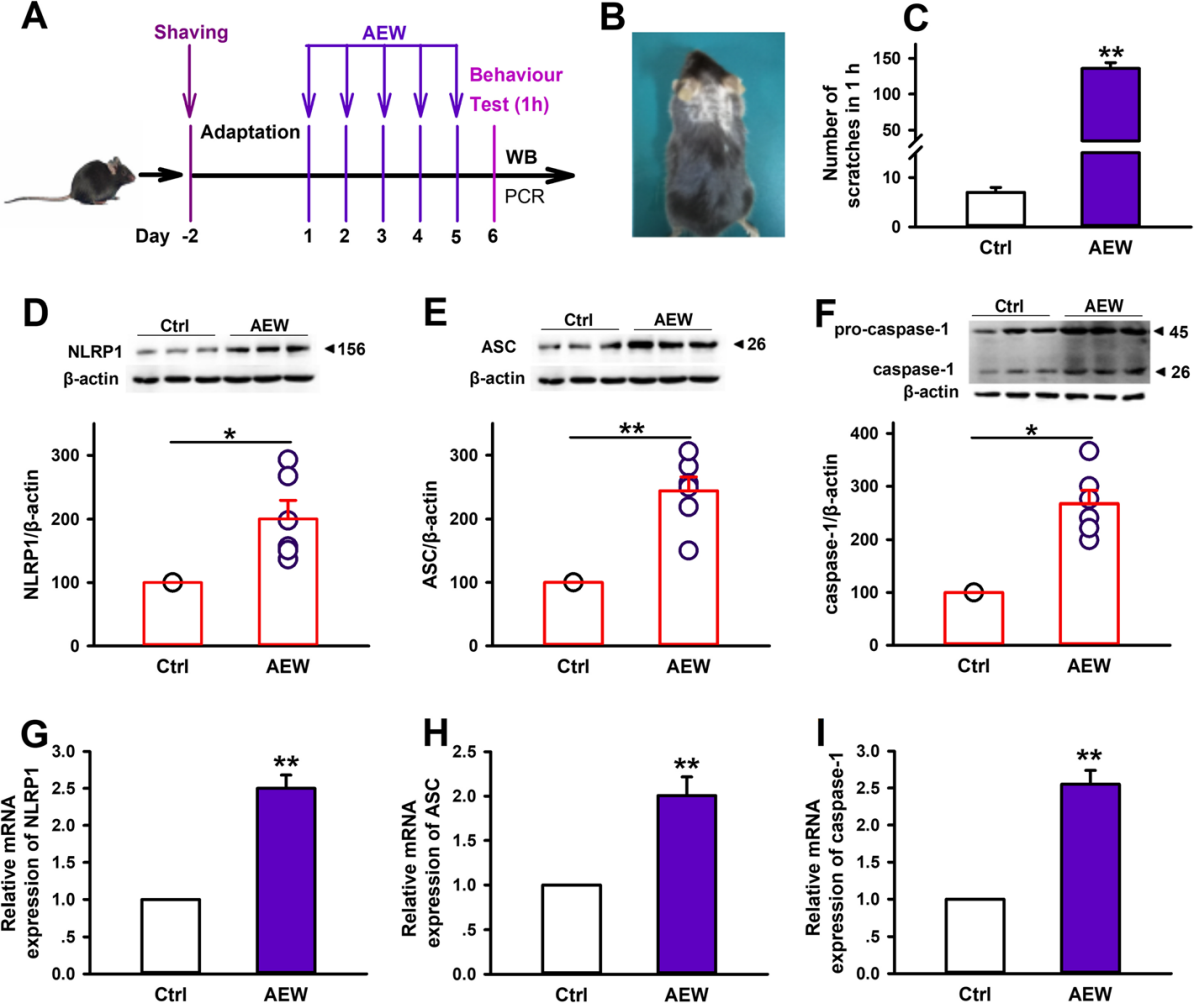
AEW treatment activates spinal cord NLRP1 inflammasome in mice. **a** The scheme of experiments with AEW-induced dry skin itch. **b** Photographs of shaved mouse back/neck after AEW treatment. **c** Statistical results showing AEW treatment increased the number of spontaneous scratches, *n* = 10. **d**–**f** Representative immunoreactive bands and statistical results showing AEW treatment increased the protein expression of NLRP1, ASC and caspase-1 in the spinal cord, *n* = 6. **g**–**i** Statistical results showing AEW treatment increased the mRNA levels of NLRP1, ASC and caspase-1 in the spinal cord, *n* = 6, Data are expressed as means ± SEM, ^*^P< 0.05 and ^**^P < 0.01 vs control group.

To investigate the role of NLRP1 inflammasome in chronic itch, we use AEW to induce skin dry itch model as previous description [35] (Fig. 1a, b). As shown in Fig. 1c, the mice developed marked scratching behavior on the sixth day after AEW treatment, suggesting chronic itch model was established successfully. Then, we test the expression of spinal cord NLRP1 inflammasome complexes in the level of protein and mRNA by western blot and real-time PCR, respectively. The results showed that the protein expression of NLRP1, ASC, and caspase-1 is significantly increased in AEW-treated group than control group (Fig. 1d–f). Similarly, compared with control group, the mRNA levels of NLRP1, ASC, and caspase-1 is also increased in AEW-treated group (Fig. 1g–i). Additionally, AEW treatment increased the expression of NLRP1 inflammasome complexes in the skin (Additional file 1). These data indicate that NLRP1 inflammasome is activated in AEW-induced chronic itch model.

### AEW treatment increases the levels of inflammatory cytokines in the spinal cord

**Fig. 2:**
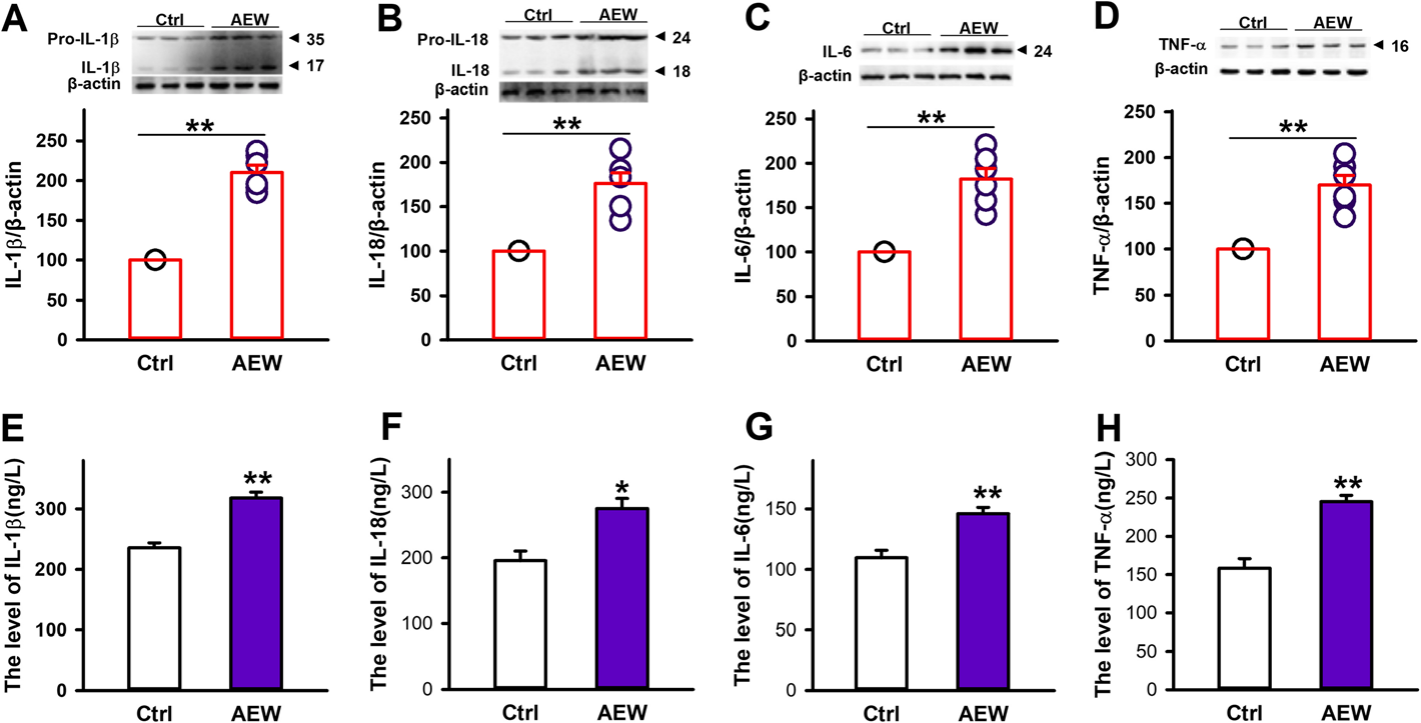
Effects of AEW treatment on inflammatory cytokines in spinal cord of mice. **a**–**d** Representative immunoreactive bands and statistical results showing AEW treatment increased the expression of IL-1β, IL-18, IL-6, and TNF-α. **e**–**h** Statistical results showing AEW treatment increased the content of IL-1β, IL-18, IL-6, and TNF-α in the spinal cord. *n* = 4. Data are expressed as means ± SEM, ^*^P < 0.05 and ^**^P < 0.01 *vs* control group

Inflammasome was considered a critical regulator of inflammatory response. Activated inflammasome can promote the maturation of IL-1β and IL-18 [11–13]. Also, inflammasome activation can stimulate the release of other inflammatory cytokines such as IL-6 and TNF-α [37]. Our results have shown that AEW treatment led to NLRP1 inflammasome activation in the spinal cord and the skin. To determine the effect of AEW treatment on pro-inflammatory cytokines, we tested the protein expression of IL-1β, IL-18, IL-6, and TNF-α by western blot. Compared with control group, AEW treatment significantly increased the expression of IL-1β, IL-18, IL-6, and TNF-α in spinal cord (Fig. 2a–d). Then, we also detected the levels of pro-inflammatory cytokines in the spinal cord by ELISA kits. Similar to above results, the levels of IL-1β, IL-18, IL-6, and TNF-α were significantly increased in AEW treatment group than control group (Fig. 2e–h). Also, AEW treatment increased the pro-inflammatory cytokines levels in the skin (Additional file 2). These results indicate that NLRP1 inflammasome-mediated inflammatory signal may be involved in AEW-induced chronic itch.

### Knockdown of Nlrp1a reduces AEW-induced scratching behavior and inflammatory effects

**Fig. 3:**
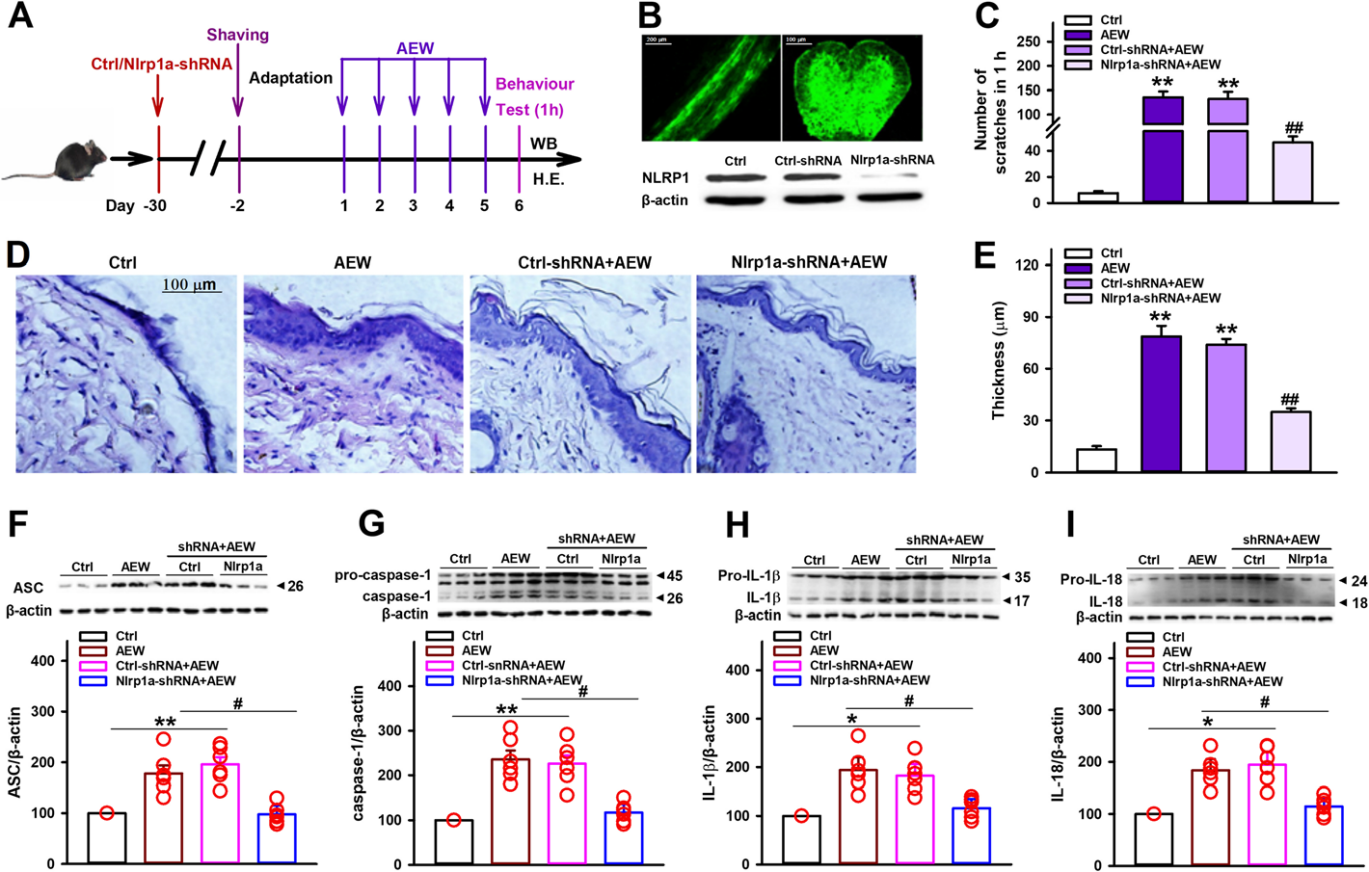
Nlrp1 knockdown inhibits AEW-induced scratching behavior. **a** The scheme of AAV-shRNA infusion and AEW-induced dry skin itch. **b** Fluorescence images that expressed AAV-Nlrp1a-shRNA-EGFP in the spinal cord 28 days after infusion. Scale bar = 200 μm for the sagittal section (left). Scale bar = 100 μm for the cross section (right). Western blotting results confirming the knockdown efficacy of AAV-Nlrp1a-shRNA, *n* = 3. **c** Statistical results showing Nlrp1 knockdown reduced AEW-induced increase in the number of spontaneous scratches, *n* = 10. **d**, **e** Representative HE staining images and statistical results showing Nlrp1 knockdown reduced AEW-induced increase in the thickness of nucleated epidermal layers, *n* = 10. Scale bar = 100 μm. **f**–**i** Representative immunoreactive bands and statistical results showing Nlrp1 knockdown reduced AEW-induced increase in the expression of ASC, caspase-1, IL-1β, and IL-18. *n* = 6, Data are expressed as means ± SEM, ^*^P < 0.05 and ^**^P < 0.01 vs control group; ^#^*P* < 0.05 vs AEW group

To test the hypothesis above, we knock down spinal cord Nlrp1a by using an adeno-associated virus (AAV) vector that selectively expresses Nlrp1a-shRNA with enhanced green fluorescent protein (AAV-Nlrp1a-shRNA-eGFP). Four weeks after AAV-shRNA infusion, the mice received AEW treatment to induce chronic itch model (Fig. 3a). As shown in Fig. 3b, Nlrp1a-shRNA showed clear silencing efficacy and significantly reduced total Nlrp1 protein levels, indicating that Nlrp1a-shRNA may be not specific and it could also targets the Nlrp1b and Nlrp1c mRNA. Compared with AEW group, control-shRNA did not influence AEW-induced scratching behavior, while Nlrp1a-shRNA significantly attenuated the number of spontaneous scratches induced by AEW (Fig. 3c). Also, AEW treatment led to a substantial increase in the thickness of nucleated epidermal layers and Nlrp1a-shRNA significantly inhibited this effect (Fig. 3d, e). Furthermore, Nlrp1a-shRNA blocked AEW-induced increases in spinal cord ASC, caspase-1, IL-1β, and IL-18 expression (Fig. 3f–i). All these results indicate that NLRP1 inflammasome-mediated inflammatory processes contribute to AEW-induced chronic itch.

### TRPV1 is involved in NLRP1 inflammasome-mediated chronic itch induced by AEW

**Fig. 4:**
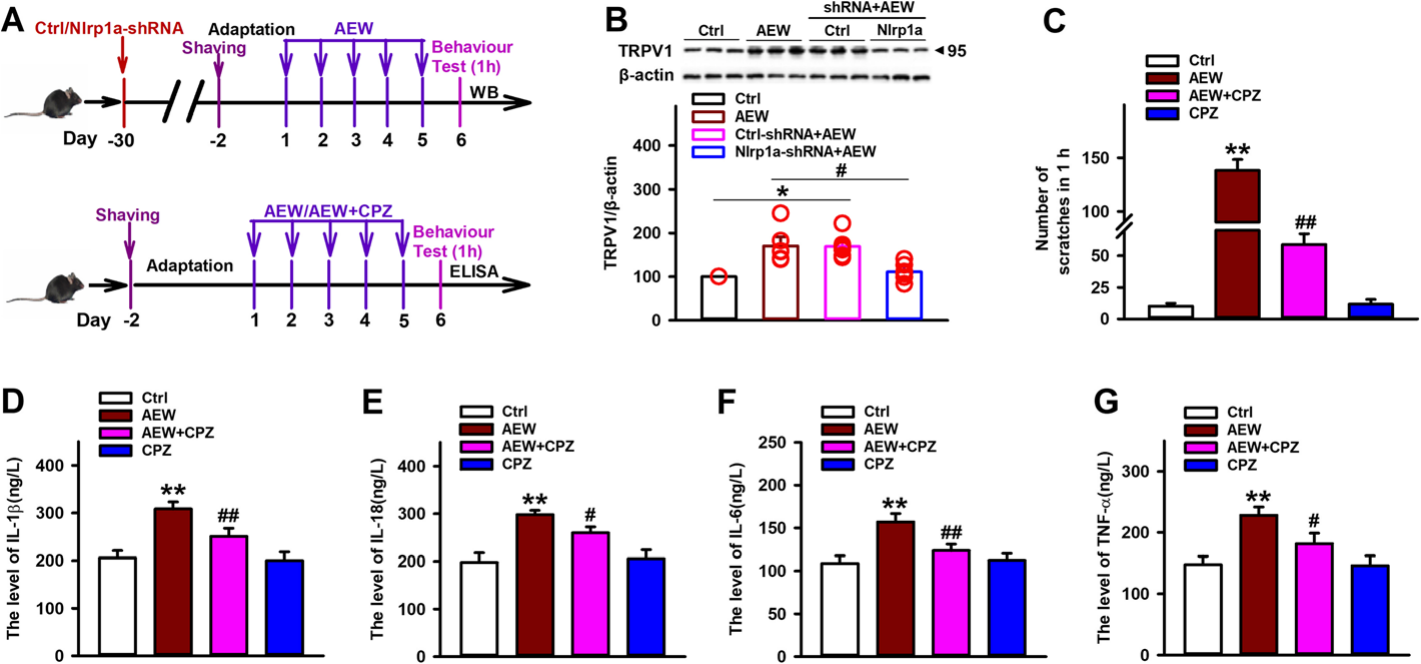
TRPV1 is involved in NLRP1 inflammasome mediated chronic itch induced by AEW. **a** The scheme of AAV-shRNA infusion, capsazepine(CPZ)treatment and AEW-induced dry skin itch. **b** Representative immunoreactive bands and statistical results showing Nlrp1 knockdown reduced AEW-induced increase in the expression of TRPV1, *n* = 6. **c** Statistical results showing CPZ (i.t., 10 μg/site) reduced AEW-induced increase in the number of spontaneous scratches, *n* = 10. **d**–**g** Statistical results showing CPZ (i.t., 10 μg/site) inhibited AEW-induced increase in the content of IL-1β, IL-18, IL-6, and TNF-α in the spinal cord, *n* = 4. Data are expressed as means ± SEM, ^*^P < 0.05 and ^**^P<0.01 vs control group; ^#^*P* < 0.05 or ^##^*P* < 0.01 vs AEW group.

TRPV1 channel, which has been reported to involve in both acute and chronic itch conditions, can be activated by pro-inflammatory cytokines and amplified the effects of inflammation [38, 39]. To investigate the mechanism that NLRP1 inflammasome is involved in AEW-induced chronic itch, we investigated the effect of NLRP1 inflammasome on TRPV1 activation in dry skin itch model (Fig. 4a). We found that AEW treatment increased the expression of TRPV1 in spinal cord. Interestingly, Nlrp1a-shRNA infusion reversed AEW-induced upregulation in TRPV1 expression, while control-shRNA has no influence on that (Fig. 4b). To further determine the role of TRPV1 in NLRP1 inflammasome-mediated chronic itch induced by AEW, the specific antagonist of TRPV1 capsazepine (CPZ, 10 μg) was intrathecally injected into the L5–L6 intervertebral space of mice 30 min before AEW treatment (Fig. 4a). Our results showed that CPZ significantly inhibited AEW-induced scratching behavior (Fig. 4c). It also inhibited AEW-induced increases in the levels of IL-1β, IL-18, IL-6, and TNF-α in the spinal cord (Fig. 4d–g). While CPZ alone has no influence on scratching behavior and inflammatory response. All these results indicate that TRPV1 channel contribute to the effect of NLRP1 inflammasome on AEW-induced chronic itch.

### NLRP1 inflammasome-mediated inflammatory signal is involved in dry skin itch in aged mice

**Fig. 5:**
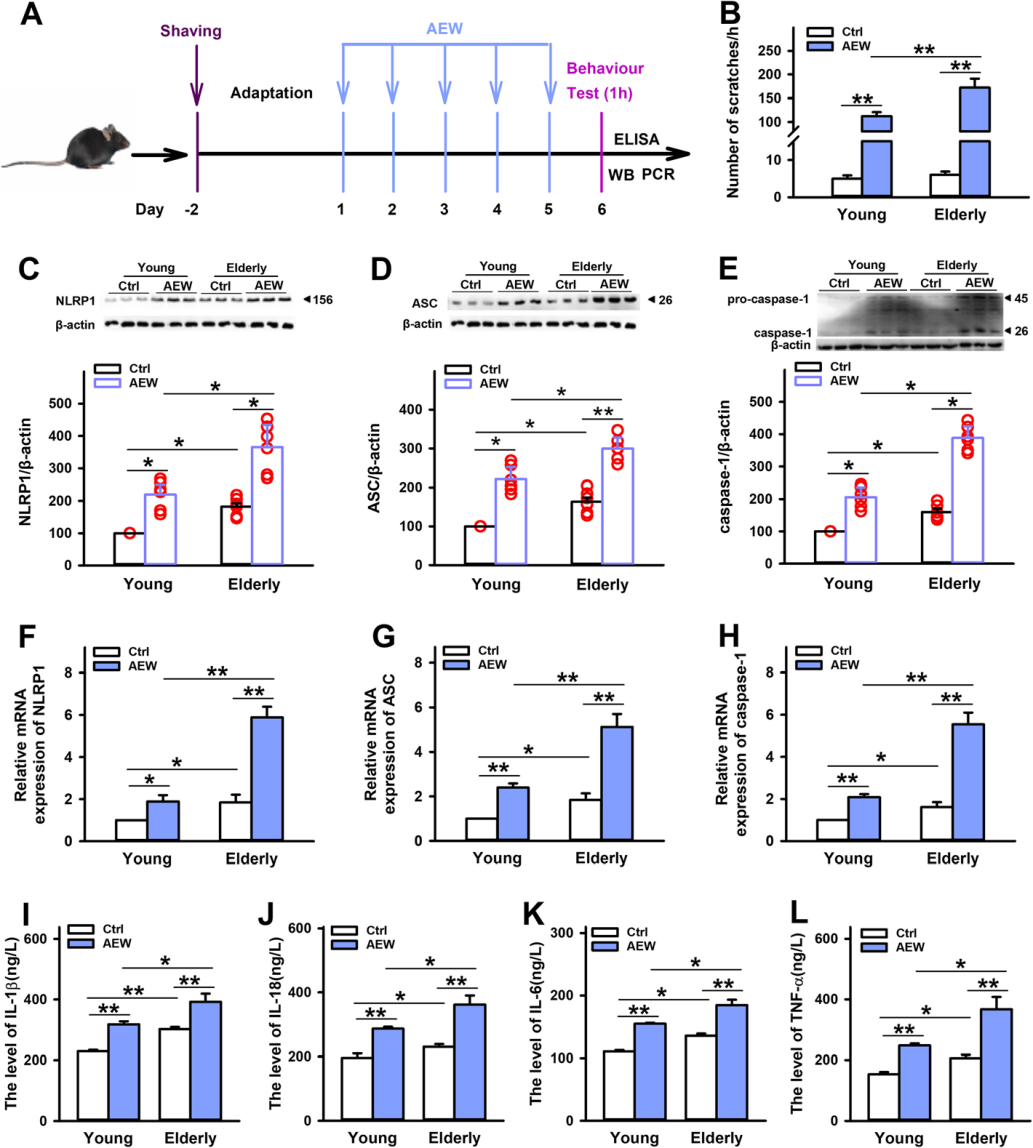
Effects of age on AEW-induced chronic itch model. **a** The scheme of experiments with AEW-induced dry skin itch. **b** Statistical results showing AEW treatment induced more significant scratching behavior in elderly mice than in young mice, *n* = 10. **c**–**e** Representative immunoreactive bands and statistical results showing AEW treatment induced more significant increases in the protein expression of NLRP1, ASC, and caspase-1 in elderly mice than in young mice, *n* = 6. **f**–**h** Statistical results showing AEW treatment induced more significant increases in the mRNA levels of NLRP1, ASC and caspase-1 in elderly mice than in young mice , *n* = 6. **i**–**l** Statistical results showing AEW treatment induced more significant increases in the content of IL-1β, IL-18, IL-6, and TNF-α in elderly mice than in young mice, *n* = 4, Data are expressed as means ± SEM, ^*^P < 0.05 and ^**^P < 0.01 vs control group or young group

Chronic itch is a common phenomenon in elderly population [32], but the correlation between aging and chronic itch has not been studied well. To further determine the role of NLRP1 inflammasome-mediated inflammatory response for dry skin itch in aged mice, AEW was used to induce chronic itch model in 20-month-old mice (Fig. 5a). As shown in Fig. 5b, compared with control group, all of young mice and elderly mice showed remarkable scratching behavior on the sixth day after AEW treatment. Moreover, elderly mice exerted more significant scratching behavior than young mice, which is consistent with previous reports. Then, we detected the expression of NLRP1 inflammasome complexes in these mice. The results showed that AEW treatment increased the expression of NLRP1, ASC, and caspase-1. Interestingly, the increases of these proteins in elderly mice were more significant than that in young mice (Fig. 5c–e). Similarly, AEW treatment increased the expressions of NLRP1, ASC, and caspase-1 in mRNA level, which was also more significant in elderly mice (Fig. 5f–h), indicating NLRP1 inflammasome may be involved in the correlation between aging and dry skin itch induced by AEW. Also, we measured the levels of inflammatory cytokines in aged and young mice after AEW treatment. As shown in Fig. 5i–l, AEW treatment increased the levels of L-1β, IL-18, IL-6, and TNF-α, which was more significant in elderly mice than in young mice. These data suggest that NLRP1 inflammasome-mediated inflammatory response is involved in enhanced chronic itch in aged mice.

### NLRP1 inflammasome-mediated inflammatory signal is involved in dry skin itch with gender difference

**Fig. 6:**
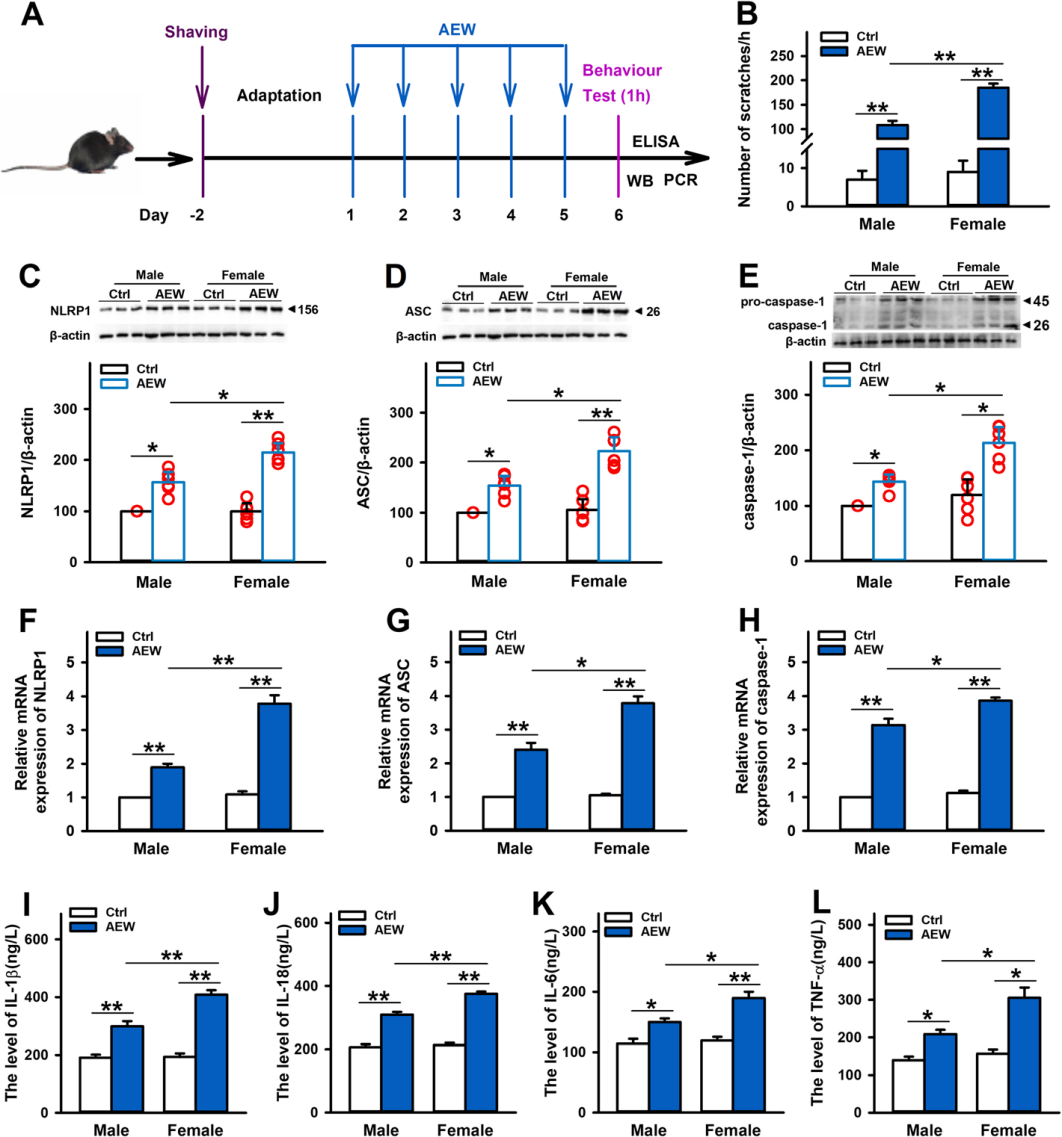
Effects of gender and sex on AEW-induced chronic itch model. **a** The scheme of experiments with AEW-induced dry skin itch. **b** Statistical results showing AEW treatment induced more significant scratching behavior in female mice than in male mice, *n* = 10. **c**–**e** Representative immunoreactive bands and statistical results showing AEW treatment induced more significant increases in the protein expression of NLRP1, ASC, and caspase-1 in female mice than in male mice, *n* = 6. **f**–**h** Statistical results showing AEW treatment induced more significant increases in the mRNA levels of NLRP1, ASC, and caspase-1 in female mice than in male mice, *n* = 6. **i**–**l** Statistical results showing AEW treatment induced more significant increases in the content of IL-1β, IL-18, IL-6, and TNF-α in female mice than in male mice, *n* = 4. Data are expressed as means ± SEM, ^*^P < 0.05 and ^**^P< 0.01 vs control group or male group

Previous studies have showed higher itch intensity ratings in women than in men [40, 41]. However, little is known about how gender relates to itch especially chronic itch. To investigate the effect of NLRP1 inflammasome-mediated inflammatory response on dry skin itch in different genders, 10-week-old mice of both sexes were used to establish chronic itch model by AEW (Fig. 6a). As shown in Fig. 6b, compared with control group, all of the male and female mice showed remarkable scratching behavior on day 6 after AEW treatment. Similar to previous reports [40, 41], higher itch intensity was observed in female mice than in male mice. Then, we further examined the expression of NLRP1 inflammasome complexes in these mice. Compared with control group, AEW treatment significantly increased the expression of NLRP1, ASC, and caspase-1 in both of protein (Fig. 6c–e) and mRNA levels (Fig. 6f–h), indicating NLRP1 inflammasome may be involved in the correlation between gender differences and dry skin itch. Also, we measured the levels of inflammatory cytokines in male and female mice after AEW treatment. As shown in Fig. 6i–l, AEW treatment increased the content of L-1β, IL-18, IL-6, and TNF-α, which was more significant in female mice than in male mice. All these data suggest that NLRP1 inflammasome-mediated inflammatory signal is involved in chronic itch with gender differences.

## Discussion

In this study, we demonstrated that spinal cord NLRP1 inflammasome-mediated inflammatory processes contribute to dry skin-induced chronic itch by activating TRPV1 channel and plays an integral role in itch transduction. Also, NLRP1 inflammasome-mediated inflammatory signal contributes to age and gender differences of chronic itch.

Chronic itch is a highly prevalent and debilitating disease which rises from various etiologies: dermatological, systemic, neuropathic, psychiatric, or other unknown origin [4, 42]. Increasing evidences showed that inflammatory cytokines and chemokines or their receptors, such as IL-31, IL-33, IL-13, IL-4, TNF-α, and CXCR3, are involved in chronic itch [43–48]. As a critical platform regulating inflammatory responses, NLRP1 inflammasome has been reported to be involved in nociception [22] and some skin diseases such as vitiligo, lupus erythematosus, pemphigus vulgaris, and psoriasis [14, 23, 24, 49, 50] Also, pro-inflammatory cytokine IL-1β and IL-18 have been reported to contribute to the development of contact hypersensitivity reactions and chronic itch [51–53]. Thus, NLRP1 inflammasome may be related to chronic itch. To test this hypothesis, AEW-induced dry skin, which is associated with several chronic itch conditions such as atopic dermatitis and xerosis [54, 55], was used to imitate the symptoms of chronic itch in mice. And the expression of NLRP1 inflammasome complexes was investigated in dry skin itch model of mice. Our current results showed that AEW treatment triggered NLRP1 inflammasome assembly and inflammatory response in the spinal cord and the skin. Whereas spinal cord Nlrp1 knockdown prevented AEW-induced scratching behavior and alleviated skin lesion, suggesting spinal cord NLRP1 inflammasome is implicated in AEW-induced chronic itch. As we know, itch is a somatosensory percept triggered by irritants at the skin’s surface. Whereas spinal interneurons receive the somatosensory by input primary afferents underlie itch, which is responsible for itch sensation. Therefore, our results indicate that NLRP1-dependent inflammation in the skin should be a triggering factor in AEW-induced chronic itch model, and that in the spinal cord is critical for the modulation of itch.

TRPV1, also called the vanilloid receptor type 1 (VR1), is one of members of the TRP super-family, is expressed in sensory neurons of the peripheral nervous system (PNS), as well as central nervous system (CNS), where it modulates the sensory transmission of nociceptive signals from the periphery [56, 57]. TRPV1 could be activated by various endogenous and exogenous stimuli, including capsaicin, temperature, pH, TNF-α, and pro-inflammatory cytokines and amplified the effects of inflammation [39, 58]. Recent studies show that dry skin stimuli increased the expression of TRPV1 in DRG and TG neurons [59, 60]. Combined with our above results, we speculate that NLRP1 inflammasome could contribute to the upregulation of TRPV1 in dry skin induced chronic itch model. As expected, our results show AEW treatment increased the expression of TRPV1 in the spinal cord, while Nlrp1 knockdown inhibited the effect. Furthermore, inhibition of TRPV1 activation with CPZ also alleviated scratching behavior and inflammatory response, indicating that NLRP1 inflammasome contributes to AEW-induced chronic itch by activating TRPV1 channel.

Aging is pertinent to physiological metabolic changes, which is inevitable for all people and has a severe impact on health systems [61]. Aging results in impaired skin barrier function, immunosenescence, and neuropathic changes, which led to skin inflammation and pruritus [62, 63]. Also, dry skin itch in elderly mice was more serious than in young mice [64]. However, little is known how age relates to chronic itch. Increasing evidences showed that age-related diseases always accompany with inflammatory process [65]. Thus, NLRP1 inflammasome may contribute to age-related chronic itch. Consistent with previous reports [64], we found that elderly mice exhibited more remarkable scratching behavior than young mice. Interestingly, the expression of NLRP1 inflammasome complexes and the related inflammatory cytokines is higher in elderly mice than in young mice. Moreover, AEW led to more significant NLRP1 inflammasome activation and higher inflammatory cytokines levels in elderly mice, indicating NLRP1 inflammasome-mediated inflammatory signal connects age to chronic itch.

In addition to age, gender is also a major factor influencing the pathological process, treatment effectiveness, and outcome of itch [41]. Women exhibit higher prevalence, higher itch intensity ratings and more burden than men [40, 41]. However, little is known about the mechanism of gender and sex differences in chronic itch. To determine whether NLRP1 inflammasome is involved in gender and sex differences in chronic itch, AEW-induced dry skin itch model was established in male mice and female mice. We found that female mice exhibited more remarkable scratching behavior, which is consistent with previous report s[40, 41]. Interestingly, the expression of NLRP1 inflammasome complexes and the related inflammatory cytokines is higher in female mice than in male mice. Nevertheless, no such changes were found between male and female control groups. These indicate that NLRP1 inflammasome-mediated inflammatory signal contributes to the gender and sex differences in chronic itch.

## Conclusions

The present study demonstrated that NLRP1 inflammasome-mediated inflammatory signal contributes to dry skin-induced chronic itch by TRPV1 channel and plays an important role in itch transduction, and it also connects age and gender to chronic itch. Therefore, inhibition of NLRP1 inflammasome may offer a new therapy for dry skin itch. However, further efforts will be made to clarify the precise mechanism how NLRP1 inflammasome affect the signaling transduction of itch in future research.

## Supplementary information

**Additional file 1: Figure S1.** AEW treatment activates skin NLRP1 inflammasome in mice. Representative immunoreactive bands and statistical results showing AEW treatment increased the protein expression of NLRP1 (A), ASC (B) and caspase-1(C)in the skin. Data are expressed as means ± SEM, n=6, *P< 0.05 and **P<0.01 *vs* control group.

**Additional file 2: Figure S2.** Effects of AEW treatment on inflammatory cytokines in the skin of mice. Statistical results showing AEW treatment increased the content of IL-1β(A), IL-18 (B), IL-6 (C) and TNF-α (D) in the skin. Data are expressed as means ± SEM, n=4, *P< 0.05 and **P<0.01 *vs* control group.

## Data Availability

The datasets used and/or analyzed during the current study are available from the corresponding author on reasonable request.

## Abbreviations

AAV: Adeno-associated virus
ACD: Allergic contact dermatitis
AD: Atopic dermatitis
AEW: Acetone-ether-water
ASC: Apoptosis-associated speck-like protein containing a caspase-activating recruitment domain
CPZ: Capsazepine
ELISA: Enzyme-linked immunosorbent assay
H.E.: Hematoxylin and eosin
NLRP1: Nucleotide oligomerization domain (NOD)-like receptor protein 1
