# Supplementary material for: Spinal cord NLRP1 inflammasome contributes to dry skin induced chronic itch in mice
Source: J Neuroinflammation. 2020 Apr 20;17:122. doi: 10.1186/s12974-020-01807-3 (PMC7168883; doi:10.1186/s12974-020-01807-3)
Supplement: Supplementary file 1 — Additional file 1: Figure S1. AEW treatment activates skin NLRP1 inflammasome in mice. Representative immunoreactive bands and statistical results showing AEW treatment increased the protein expression of NLRP1 (A), ASC (B) and caspase-1(C)in the skin. Data are expressed as means ± SEM, n=6, *P< 0.05 and **P<0.01 vs control group. [file 12974_2020_1807_MOESM1_ESM.pdf]

Figure S1.

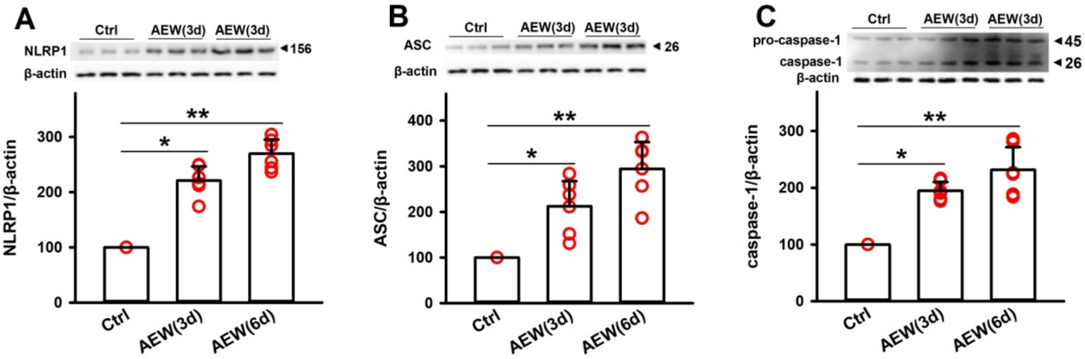

Fig S1. AEW treatment activates skin NLRP1 inflammasome in mice. Representative immunoreactive bands and statistical results showing AEW treatment increased the protein expression of NLRP1 (A), ASC (B) and caspase-1（C） in the skin. Data are expressed as means ± SEM, n=6, $^{*}P< 0.05$ and $^{**}P<0.01$ *vs* control group.
