# Supplementary material for: Spinal cord NLRP1 inflammasome contributes to dry skin induced chronic itch in mice
Source: J Neuroinflammation. 2020 Apr 20;17:122. doi: 10.1186/s12974-020-01807-3 (PMC7168883; doi:10.1186/s12974-020-01807-3)
Supplement: Supplementary file 2 — Additional file 2: Figure S2. Effects of AEW treatment on inflammatory cytokines in the skin of mice. Statistical results showing AEW treatment increased the content of IL-1β(A), IL-18 (B), IL-6 (C) and TNF-α (D) in the skin. Data are expressed as means ± SEM, n=4, *P< 0.05 and **P<0.01 vs control group. [file 12974_2020_1807_MOESM2_ESM.pdf]

Figure S2.

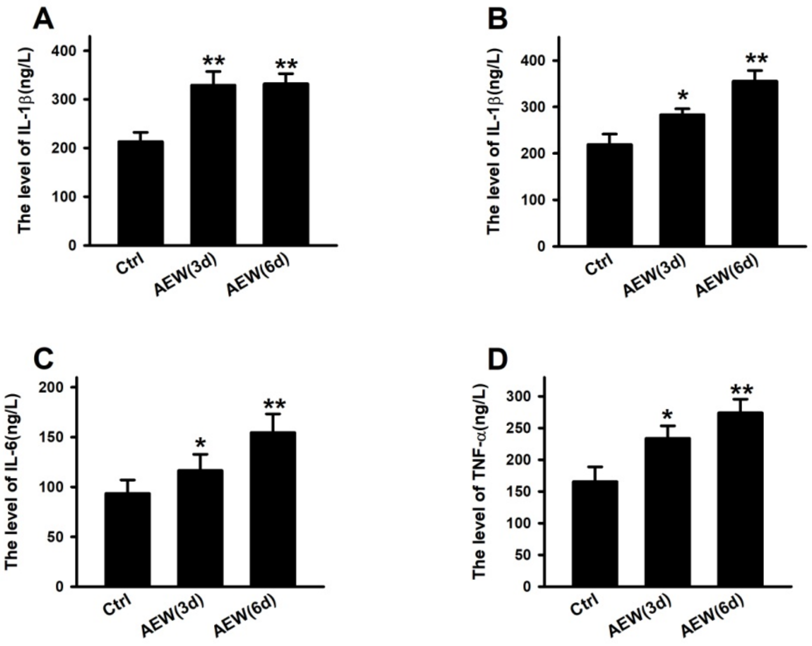

Fig S2. Effects of AEW treatment on inflammatory cytokines in the skin of mice. Statistical results showing AEW treatment increased the content of IL-1β（A）, IL-18 (B), IL-6 (C) and TNF-α (D) in the skin. Data are expressed as means ± SEM, n=4, $^{*}P< 0.05$ and $^{**}P<0.01$ *vs* control group.
